# Metabolism Characteristics of Th17 and Regulatory T Cells in Autoimmune Diseases

**DOI:** 10.3389/fimmu.2022.828191

**Published:** 2022-02-25

**Authors:** Yan Qin, Chong Gao, Jing Luo

**Affiliations:** ^1^Department of Rheumatology, The Second Hospital of Shanxi Medical University, Taiyuan, China; ^2^Department of Pathology, Brigham and Women’s Hospital, Harvard Medical School, Boston, MA, United States

**Keywords:** immunometabolism, Th17 cells, regulatory T cells, autoimmune disease, single-cell metabolism, gut microbiota

## Abstract

The abnormal number and functional deficiency of immune cells are the pathological basis of various diseases. Recent years, the imbalance of Th17/regulatory T (Treg) cell underlies the occurrence and development of inflammation in autoimmune diseases (AID). Currently, studies have shown that material and energy metabolism is essential for maintaining cell survival and normal functions and the altered metabolic state of immune cells exists in a variety of AID. This review summarizes the biology and functions of Th17 and Treg cells in AID, with emphasis on the advances of the roles and regulatory mechanisms of energy metabolism in activation, differentiation and physiological function of Th17 and Treg cells, which will facilitate to provide targets for the treatment of immune-mediated diseases.

## Introduction

In T cell biology, immunometabolism is intrinsically linked to cellular development, activation, function, differentiation and survival (1, 2). In 2002, immunometabolism, a new branch of metabolism was came to light with the discovery of the link between CD28 activation and glycolysis in T cells (3). Metabolic processes can provide energy for T cells as well as acting as regulators of T cell differentiation (4, 5). In recent years, accumulating evidences indicated that the cellular metabolism coordinates activation, differentiation and function of T cell, and governs the final outcome of T cell-mediated immune response and autoimmune diseases (AID) (6, 7).

Naïve T cells are maintained in a state of hyporesponsiveness called quiescence, which is characterized by small cell size, low proliferative rate, and low basal metabolism. Quiescent T-cell energy demand is low, without the need to obtain the energy rapidly, and they generate energy mainly through oxidative phosphorylation (OXPHOS) in the mitochondria and breakdown of fatty acids. Differentiated and activated T cells engage in a metabolic profile absolutely distinct from that of their quiescent counterparts and undergo metabolic reprogramming in order to provide energy for clonal expansion and effector function in a short period of time (2, 8, 9). Firstly, the glucose transporter (Glut) and Alanine–Serine–Cysteine Transporter (ASCT2), glutamine transporters, are highly expressed (10, 11). Meanwhile, mammalian targets for rapamycin (mTOR), a serine/threonine kinase and an crucial regulator of the cellular metabolism, acts on Myc and hypoxia-inducible factor-1 (HIF-1α), shifting T cell metabolism from pyruvate oxidation and fatty acid oxidation (FAO) to glycolysis, glutamine hydrolysis, and pentose phosphate pathways (PPP), thereby promoting T cell differentiation and proliferation (8, 12).

The subtle differences in metabolic reprogramming of quiescent T cells will lead naive CD4+T cells to differentiation into different T cell lineages, including crucial immune effector T help 17 subset (Th17) and immunosuppressor regulatory T subsets (Tregs), which are capable to influence a variety of immune cells and are essential for controlling inflammation and preventing from attacking self-antigens (13). Th17/Treg cell imbalance has been widely ascribed as the key factor for the autoimmune pathology in patients with rheumatoid arthritis (RA), systemic lupus erythematosus (SLE), sjogren’s syndrome (SS) (14). Thus, understanding the metabolic process and functional heterogeneity of Th17 and Treg cell development may offer new therapeutic targets and approaches for the cure of AID.

## Reciprocal Polarisation of TH17 and TREG Cells

Naïve CD4+ T cells differentiate into distinct effector lineages as a consequence of the interaction between antigen and specific cytokine signals. Before the elucidation of Th17 cells as a unique CD4+T lymphocyte subset, which were classified into two major subsets: Interleukin (IL)-2 and interferon (IFN)-γ-secreting Th1 and IL-4 and IL-5-secreting Th2 cells. IL-17, the signature cytokine of Th17 cells, was described in 2000 and was later shown to induce joint damage in murine models of arthritis (15). IL-17A is one of the IL-17 family members that stimulates epithelial, endothelial, and fibroblastic cells to secrete pro-inflammatory factors such as IL-6, IL-8, granulocyte-macrophage colony stimulating factor (GM-CSF), chemokines (CXCL1, CCL20) as well as prostaglandin E2 (16, 17). Subsequently, it was proposed that IL-17-producing Th17 cells represent a novel CD4+ T cell subset which is different from Th1 and Th2 subsets and IL-23 promotes the development and expansion of Th17 cells (17, 18). Nevertheless, the differentiation of naive T cells into Th17 is not only dependent on IL-23, but also requires the co-drive of IL-6 and transforming growth factor-beta (TGF-β) during the initial phase. TGF-β and IL-6 induce retinoid acid-associated orphan receptors (RORs) by activating transducer and activator of transcription (STAT3). RORγt further regulates the transcription of IL-17, IL-21 and IL-22, all of which are Th17-specific genes (19). In addition, IL-21 has been identified as an important cytokine in growth and amplification of Th17 cells (20). IL-1β is another important pro-inflammatory factor that induces Th17 cell differentiation, mainly through activation of the mTOR pathway (21, 22). mTOR activates catabolic pathways, especially aerobic glycolysis, and induces fatty acid synthesis (FAS), promoting TCR-mediated naive T cells to differentiate into Th17 cells (23).

On the other hand, the capacity of CD4+T cell subset to suppress immune responses was first described in the early 1970s (24). Initially the anti-inflammatory capacity of CD4+T cells were described as one population of peripheral T cells, which co-express the interleukin-2 receptor (IL-2R) alpha-chain (CD25) by Sakaguchi in 1995 (25). Whereas, the utility of CD25 expression as a Treg marker is limited since it is also an activation marker for lymphocytes and does not discriminate between activated T effector cells and Tregs. The CD4+CD25+CD127-/low population was thought to be a better approach for the identification of human Treg cells in a while. More recently, studies have confirmed Treg cells highly express the forkhead box P3 (FOXP3), which plays a key role in the development and function of Treg cells and becomes a more specific marker for Treg cells (26). FOXP3, as a nuclear transcription factor, is mainly localizes in the nucleus and participates in the regulation of TCR signal transduction, cell communication and transcription through interaction with other transcription factors (27). In addition, some markers such as CTLA-4, CD45RA and CD45RO were used for fine typing of Treg cells in humans (28, 29). Treg cells use different pathways to exert suppress function: an intercellular contact dependent mechanism *in vitro*; *in vivo* regulatory function mainly depends on immune modulatory cytokines, such as IL-10, IL-4, IL-35 and secretory TGF-β; interacting with antigen-presenting cells and metabolic disturbance of target cells (30). Increasing evidences show that human autoimmune diseases are associated with impaired suppressive function and/or decreased number of Treg cells (31–34).

**Figure 1:**
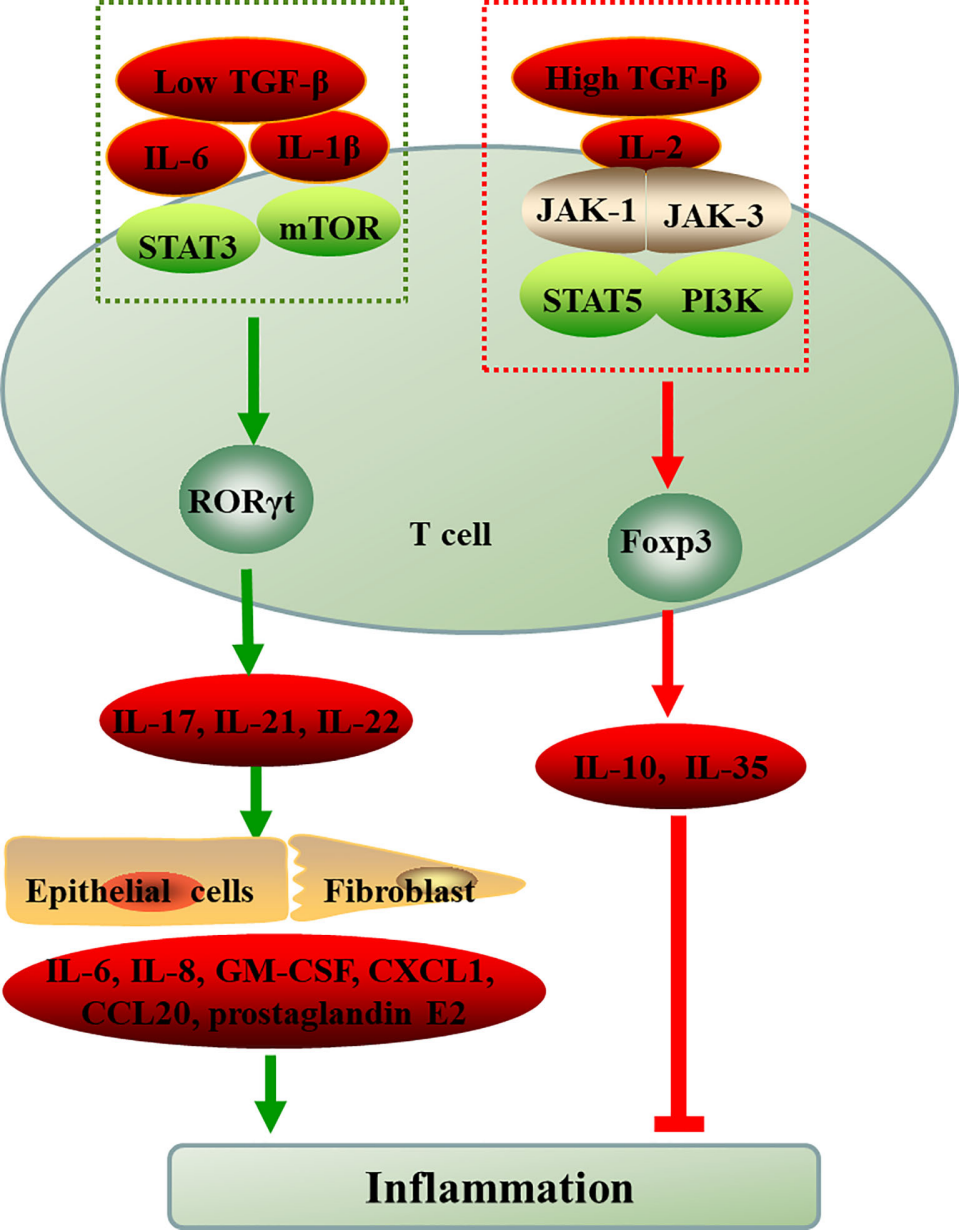
The differentiation of naive CD4+T cell into Th17 and Treg cells. ROR-γt and Foxp3, the respective lineage-specific transcription factors for Th17 and Treg cells. IL-6 or IL-1β induce the differentiation of T cells into Th17 cells through STAT3 or mTOR in the presence of low TGF-β, however, IL-2 critically drives high TGF-β-treated CD4+ cells to differentiate into Foxp3+ Treg cells *via* JAK-STAT. The inflammatory environment controls the balance between Treg and Th17 cell differentiation cellular metabolism controls T cell lineage choices. TGF-β, transforming growth factor-beta; IL, interleukin; JAK, Janus kinase; STAT, signal transducer and activator of transcription; mTOR, mammalian target of rapamycin; PI3K, phosphatidylinositol 3-kinase; RORγt, retinoid-related orphan receptor-γt; Foxp3, forkhead box P3; Th, T-helper cell; Treg, regulatory T cell; GM-CSF, granulocyte-macrophage colony stimulating factor; CXCL1, chemokines; CCL20, chemokines.

ROR-γt and Foxp3, the respective lineage-specific transcription factors for Th17 and Treg cells. Despite their different functions in tolerance and inflammation, both Th17 and Treg cells can develop from naive CD4+T cell precursors under the influence of the TGF-β. IL-6 or IL-21 induce Th17 cells through transcription factor orphan nuclear receptor RORγt exclusively drives TGF-β-treated T cells to become Th17 cells, however, IL-2 critically drives TGF-β-treated CD4+ cells to differentiate into Foxp3+ Treg cells (35). In addition, in the presence of high TGF-β concentrations, the cells cannot differentiate into Th17 cells and eventually develop into Foxp3+ Treg cells. However, low concentration TGF-β combined with IL-6 and IL-21 impaired Foxp3 activity and promoted Th17 cell development (36, 37). The binding of cytokines and receptors initiates complex intracellular intermolecular interactions that trigger signal cascade amplification and ultimately lead to changes in gene transcription of specific cell-effector molecules. Thus, cytokines and environment cues play pivotal roles in the differentiation, development and function of Th17 and Treg (**Figure 1**). mTOR, a sensor for environmental cues, senses the immune microenvironment and regulate cellular metabolism to direct T cell differentiation (38).

## Multiple Metabolic Pathway Regulate TH17/TREG Balance

### mTOR Dependent Glycolytic Pathway Orchestrates the Th17/Treg Balance

It has been well appreciated that the bioenergetic demands of T cells increase dramatically over the resting state upon antigen stimulation. Metabolic remodeling from OXPHOS to aerobic glycolysis is a hallmark of T cell activation and is thought to be required to meet the metabolic demands of proliferation (12). The glycolysis not only expedited production of ATP (100 times faster), but also provided necessary intermediates for other metabolic pathways, such as PPP, hexosamine biosynthesis, amino acid biosynthesis, and fatty acid synthesis (FAS) (39). Recently, a role for the metabolic pathways in T cell activation and the differentiation of T cell functional subsets is beginning to be appreciated. Study demonstrated that cellular metabolism controls T cell lineage choices.

**Figure 2:**
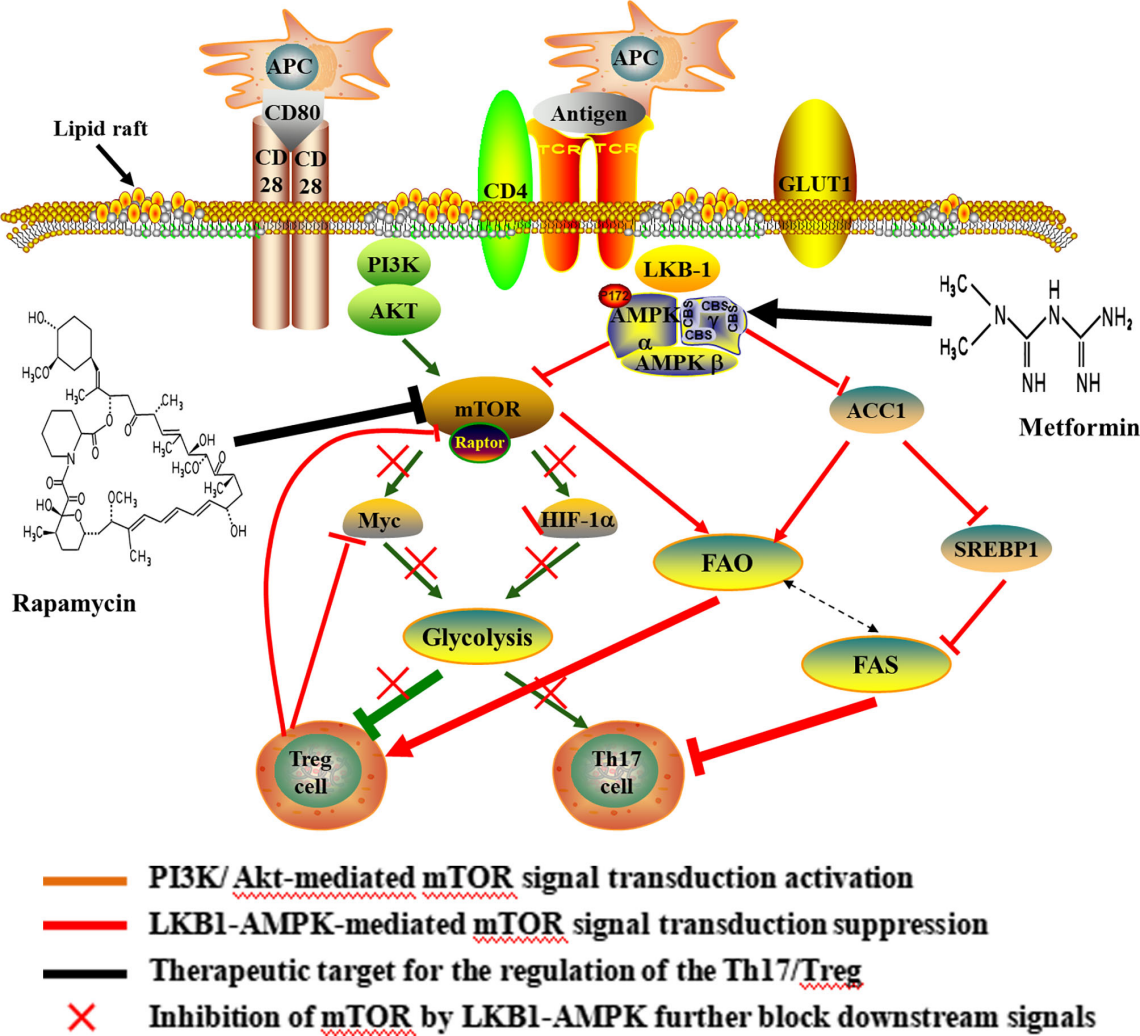
Potential mechanisms and therapeutic target of Th17/Treg metabolic homeostasis. Upon antigen stimulation, cellular metabolism controls T cell lineage choices. During T cell activation, CD28 up-regulates glycolysis by promoting the increase of c-myc and the Glut1 in CD4+ T cells. The increase of glycolysis induced by CD28 was important for differentiating of Th17 cells. The activation of mTORC1 is mainly mediated by the PI3K/Akt and LKB1-AMPK, thus enhancing glycolysis and predisposing naïve T cells to differentiate into inflammatory Th17 cell. Over-activation of mTOR, Myc and HIF1 promote glycolysis and weaken suppressive activity of Treg cell, and ultimately lead to the loss of suppressive function in inflammatory conditions. In turn, Foxp3 inhibited c-Myc in Treg cells. Also, the lipid metabolism affected the differentiation of CD4+ T cells, and the differentiation of Th17 cells required FAS, but Treg cell was rely on FAO. Inhibition of mTOR can result in the elevated FAO and promote Treg differentiation. In addition, rapamycin-mediated inhibition of mTOR blocks glycolytic activity and increases the FAO. Metformin, an AMPK agonist, which exerts anti-inflammatory effects on CD4+ T cells and induce Treg expansion and lipids alterations. The use of rapamycin and metformin lead to pro-oxidative effects following CD4+T cell stimulation. APC, antigen presenting cell; CD, cluster of differentiation; GLUT, Glucose transporter; AKT, proteinserine-threonine kinase; LKB-1, liver kinase B-1; AMPK, Cyclic Adenosine monophosphate-activated protein kinase; HIF-1α, hypoxia-inducible factor-1; ACC, acetyl-CoA carboxylase; SREBP1, sterol regulatory element-binding protein 1; FAO, fatty acid oxidation; FAS, fatty acid synthesis.

As a hub in the cellular metabolic signal network and sensor for environmental cues, mTOR controls multiple T cell lineage fates and functions, including Th17 and Treg cells (40–43) (**Figure 2**). The mTOR works by forming two complexes (mTORC1 and mTORC2) with a variety of proteins. mTORC1 and mTORC2 share the catalytic subunit mTOR but are distinguished by the scaffold proteins regulatory associated protein of mTOR (RAPTOR) and rapamycin-insensitive companion of mTOR (RICTOR), respectively (41). The mTOR complexes have been shown to be differentially expressed in murine effector T cells. Th17 and Th1 cells mostly express mTORC1, while Th2 cells predominantly express mTORC2 (44). mTORC1 is known to inhibited by AMP activated protein kinase (AMPK), which is regulated by liver kinase B1 (LKB1). In addition, mTORC1 promote Th17 differentiation by PI3K/AKT pathway and to promoting Glut1 cell-surface trafficking and glycolysis (45–47). As outlined above, the activation of mTORC1 signaling, either through PI3K/Akt-mediated signal transduction or suppression of LKB1-AMPK activity, enhance glycolysis and predisposed naïve T cells to differentiate into inflammatory Th17 cell (48, 49). mTOR, on the flip side, is critically required for Treg functional in mice (50, 51). Murine Treg cells have been shown to express less mTORC1 (52). Over-activation of mTORC1 promotes glycolysis, destabilizes Tregs and impairs their suppressive activity, leading to the loss of suppressive function in inflammatory conditions (45). Withal, FOXP3 expression inhibited mTORC1 activity and glycolysis, and enhanced mitochondrial oxidative (53). In a word, mTOR dependent glycolysis promotes Th17 development while hampering Treg cell formation. Study has demonstrated that rapamycin can blocks mTOR-dependent metabolic pathways including glycolytic activity and the production of IL-17 (54).

HIF1α is downstream of mTOR signaling, thereby playing an important role in regulating mTOR-mediated T cell differentiation (42, 55). Study demonstrated that HIF1α was selectively expressed in Th17 cells and regulates the expression of multiple glycolytic enzymes (55). Murine studies have shown a clear role for metabolism in reciprocally regulating the *de novo* differentiation of Th17 and Treg cells where HIF1α promote Th17 but to inhibit Treg cell differentiation when naive T cells were activated in the presence of TGF-β (52, 56). Deficiency in HIF1α resulted in greatly reduced glycolytic activity, and results in impairment in the Th17 differentiation program, but increased development of Tregs (54). Therefore, the HIF-1α-dependent glycolysis pathway is critical for regulating Th17 and Treg cell differentiation.

Myc is another critical regulator in T cell metabolism and a potential downstream target of mTOR (8, 57). During T cell activation, Myc as a transcription factor for GLUT1 (the main glucose transporter in lymphocytes), hexokinase 2 (HK2), pyruvate kinase (PK), and lactate dehydrogenase A (LDHA) induces glycolysis. Study has indicated that the pyruvate kinase muscle isozyme (PKM2) inhibitor suppresses glycolysis and the differentiation of Th1 and Th17, thereby reducing disease activity and alleviating experimental autoimmune encephalomyelitis (EAE) by inhibiting PK activity (58). Therefore, down-regulation of the transcription factorc-Myc is critical in inhibiting glycolysis (8). Also, Myc can be rapidly translocated to the plasma membrane through the Akt signaling pathway, thereby increasing glycolysis. Study has demonstrated that CD28 up-regulates glycolysis independent of the T cell receptor (TCR) engagement by increasing the Glut1 and c-myc in CD4+ T cells in patients with multiple sclerosis, which was critical for secreting inflammatory cytokines of Th17 cells (59). Additionally, Foxp3 inhibited c-myc in Treg cells, which was relevant to the impaired glycolysis and the enhanced oxidative phosphorylation (60). Collectively, the mTOR dependent glycolytic process is a metabolic checkpoint for T cell lineage choices and alters Th17 and Treg cell differentiation. Furthermore, studies indicated that rapamycin undertook the role in the survival and proliferation of Treg cell (61, 62). Recent research revealed that rapamycin recovery immune tolerance by intervening with metabolic reprogramming of Treg cells (63). The mTOR dependent glycolytic pathway has provided targets for therapeutic intervention of autoimmune and inflammatory diseases elicited by T cells.

### Lipid Metabolism Driving Th17 and Treg Differentiation

Lipid metabolism is another important pathway by which immune cells can potentially be modulated (**Figure 2**). The development of Th17 cells was dependent on FAS, whereas differentiation of Treg cell required FAO (52, 64). It is well known that free fatty acids (FFA) in cytoplasm are first catalyzed by acyl coenzyme A synthetase (ACS) to produce fatty acyl CoA, then is transported into the mitochondria to produce acetyl-CoA by carnitine palmityl transferase (CPT)-1, CPT-2 and translocase and enter the tricarboxylic acid cycle (TCA). Murine study has demonstrated that Treg cell differentiation was inhibited following treatment with inhibitor of CPT1, while no effect on the differentiation of Th17 cell (52). Additionally, acetyl-CoA carboxylase (ACC) plays an important role in FAS and catabolism, and is a key enzyme catalyzing fatty acid anabolism. In humans and other mammals, it is a tissue-specific enzyme and exists in two gene forms, ACCI and ACC2. ACC1 exists in the cytoplasm of most adipose tissues (liver, adipose) and catalyzes rate-limiting reaction of long chain fatty acid (LCFA) synthesis. ACC2 is distributed in the heart and muscle tissue, and there are about 140 more amino acids than ACCI at the N-terminal. These amino acid sequences promote ACC2 to anchor on the mitochondrial outer membrane, thus catalyzing the oxidation of mitochondrial fatty acids (65). In the presence of ATP and Mg2+, HCO- as carboxyl donor, acetyl-CoA is carboxylated into malonyl monoacyl-coA by ACC, which plays a central role in Th17 and Treg cell differentiation by regulating *de novo* FAS. Inhibiting ACC using soraphen, a specific inhibitor of ACC isozymes, under Th17 polarizing conditions skews differentiation towards Tregs in mouse (64). This study also demonstrated that inhibition of ACC has the same effect on the differentiation of human T cells (64). Meanwhile, AMPK can indirectly inhibit FAS by hampering sterol regulatory element-binding protein 1 (SREBP1) and inactivating ACC1, thus inhibiting Th17 cell (66, 67).

On the other hand, FAO is generally associated with an anti-inflammatory phenotype of Treg cells and plays a vital role in maintaining the stability of Treg lineage. In murine studies, newly differentiated Treg cells demonstrated the highest the FAO compared with other effector T cell subsets. Blocking FAO significantly reduced the differentiation of Treg cell and downregulated its immunosuppressive function. Treg cells exhibited elevated phosphorylation and activation of AMPK, which is known to promote mitochondrial lipid oxidation and could give rise to increased FAO while inhibiting mTOR (8, 68, 69). In turn, inhibition of mTOR can lead to increased oxidation of exogenous FA, thus promoting Treg differentiation (70, 71). Furthermore, study have demonstrated that promoting FAO makes the Th17/Treg differentiate in favor of Treg cells, and inhibition of FAS can significantly reduce the ratio of Th17/Treg cell in the absence of glycolysis (72). During initial stage of T cell activation, supplement of lipids intensely restrain the production of Th17 cytokines, but not Treg suppressive function (52). Intriguingly, the up-regulation of FAS associates with down-regulation of FAO, which manifests that metabolic pathways interconnected (8). Collectively, the lipid metabolism affected the differentiation of CD4+ T cells, and inhibition of FAS or promotion of FAO modulates the Th17/Treg axis in favor of Treg cells.

Metformin, an AMPK agonist, which exerts anti-inflammatory effects on CD4^+^ T cells and induce Treg expansion and lipids alterations in various models of autoimmune disease, including experimental autoimmune encephalomyelitis and arthritis (73, 74). In the murine study, enhancing FAO *via* AMPK activation using the AMP analog 5-aminoimidazole-4-carboxamide ribonucleotide (AICAR), promoted the polarization of Treg cells and inhibited Th17 cells both *in vitro* and *in vivo* (75). FAO also plays a crucial role in maintaining the functional stability of Treg cells. Foxp3 extend its half-life by preventing from targeted degradation through post-transcriptional modifications, such as acetylation. Foxp3 acetylation is mainly dependent on acetyl-CoA, whose supply is increased upon breakdown of fatty acids (76). In addition, Rapamycin-mediated inhibition of mTOR increases the rate of FAO in skeletal muscle cells both *in vivo* and *in vitro* while decreasing flux into anabolic storage pathways by decreasing level of the enzyme, including ACC1, malonyl-CoA and glycerol phosphate acyltransferase (70, 77). These studies suggest that inhibition of mTOR and activation of AMPK play a physiological role in the regulation of fatty acid metabolism and resulted in prooxidative effects following CD4^+^T cell stimulation.

### IL-2/IL-2R Regulates the Metabolism of Treg and Th17 Cells

Cell-intrinsic metabolic pathways directly impact cellular fate and function. IL-2 as a pleiotropic cytokine, is a key regulator of T cell metabolic programs and is critical for the development of Tregs in the thymus and the regulation, proliferation, and maintenance of Tregs in peripheral tissues (78, 79). It has been demonstrated that the IL-2 critically drives TGF-β-treated CD4+ cells to differentiate into Foxp3+ Treg cells (79, 80). It is well known that high-affinity IL-2 receptor (IL-2R) is composed of three subunits: CD25 (the IL-2 receptor α chain, IL-2Rα), CD122 (IL-2 receptor β chain, IL-2Rβ) and CD132 (common γ subunit, γc) (81, 82). The co-expression of all three subunits is needed to confer high-affinity IL-2 binding to a cell. The signaling pathways that IL-2/IL-2R control the differentiation and homeostasis of both pro- and anti-inflammatory T cells is fundamental to determining the molecular details of immune regulation (83).

**Figure 3:**
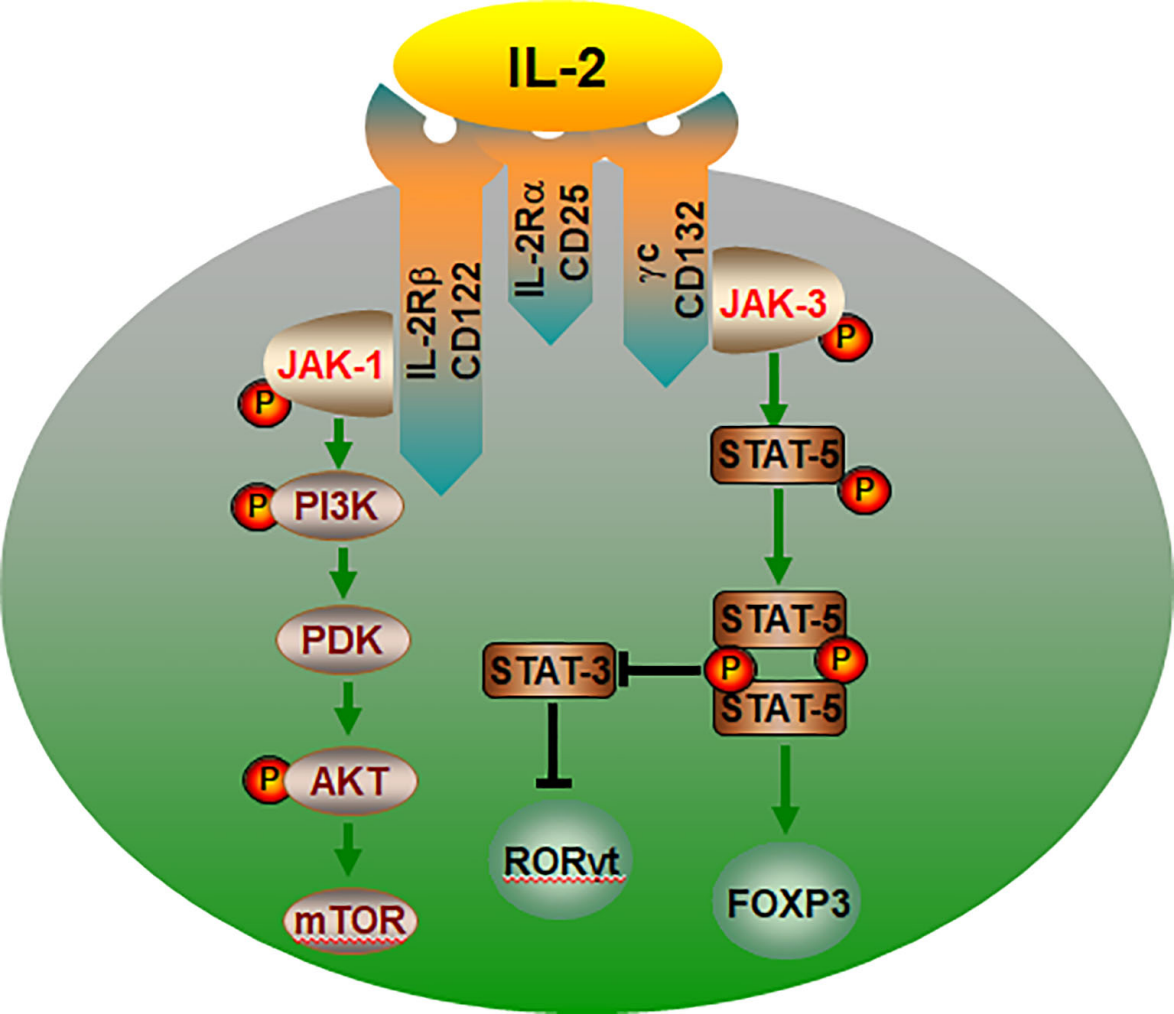
The IL-2/IL-2R signaling pathways control the lineage choice of Th17 and Treg cells. The co-expression of IL-2Rα, IL-2Rβ and γc is essential for IL-2 binding to cell. IL-2 regulation of Treg cells is mediated by the PI3K-Akt and JAK-STAT5 pathway. JAK-STAT5 pathway promotes Treg cells differentiation, and inhibiting transcription of the IL-17, thereby suppressing Th17 cell. In addition, IL-2 can activate mTOR through PI3K-AKT and promote the differentiation of CD4+ T into Th17 cells. IL-2R, interleukin-2 receptor; P, phosphorylate; PDK, phosphoinositide-dependent kinase.

It is accepted that IL-2 activates two major signaling axes: the STAT5 and PI (3)K pathways (84, 85) (**Figure 3**). IL-2 activation of STAT5-mediated transcriptional programs is vital to the biological actions of IL-2 and is particularly important for Treg development, as it is necessary to initiate Foxp3 expression (86–89). A mice study showed that deficient of STAT5A/B fail to generate Tregs, demonstrating that the crucial role for IL-2-STAT5 in Treg development (90). In humans, loss of function in STAT5B is also associated with a loss of Tregs (91). Furthermore, in Stat5a/b^fl/fl^ mice, the deletion of the transcription factor STAT5 resulted in enhanced Th17 cell development when the absence of IL-2 or disruption of its signaling (92). STAT5 binding to the IL17a-IL17f locus inhibiting STAT3- mediated transcription of the IL17 gene and thereby suppressing Th17 cell differentiation (93). Another key signaling pathway in IL-2 regulation of Treg cells is mediated by the PI3K-Akt (94). And its biological effects could be mediated by the activation of cytosolic serine/threonine kinases. As an important serine/threonine kinase, mTOR senses various environmental cues, such as IL-2 (95, 96). Whereas IL-2 control of mTORC1 activity is dependent on JAK kinase activity. The IL-2Rβ chain binds JAK1, the IL-2Rγc binds to JAK3 (97). Thus, IL-2 receptor occupancy results in JAK-STAT activation (98). In addition, IL-2 regulates the expression and activation of MYC, mTORC1 and HIF1α/HIF1β, thereby guaranteeing the uptake of glucose (99). Recent studies have confirmed that the Treg dysfunction in the pathogenesis of AID can be restored by IL-2 effects of IL-2 (32, 34, 100–102).

### The Gut Microbiota at the Service of Immunometabolism of Th17/Treg Differentiation

**Figure 4:**
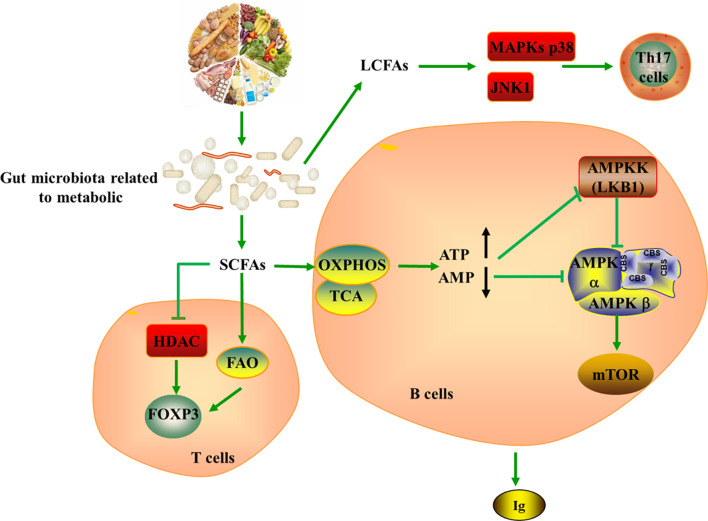
Tying microbiota metabolism to Th17/Treg balance. SCFAs produces by gut microbiota metabolizing dietary fiber can serve as a substrate for FAO, further producing acetyl-CoA, which provides fuel for TCA and OXPHOS. SCFAs strengthen the extrathymic generation of Treg cells by inducing Foxp3 in HDAC-dependent mode, while LCFAs promote the generation of pathogenic Th17 cells *via* the MAPKs p38 and JNK1. In addition, SCFAs could indirectly induce the production of ATP and the depletion of AMP, leading to the activation of mTOR in B cell and the generation of immunoglobulin. SCFAs, short-chain fatty acids; LCFAs, long-chain fatty acids; ATP, Adenosine-triphosphate; AMP, Cyclic Adenosine monophosphate; HDAC, histone deacetylase; MAPKs, mitogen-activated protein kinase; JNK, c-Jun N-terminal kinase; AMPKK, AMPK kinase; TCA, tricarboxylic acid cycle; OXPHOS, oxidative phosphorylation.

The majority of microorganisms present in our body are resided in the human gut, affecting the balance between pro- and anti-inflammatory immune responses (103). The prominent role of the gut microbiota in the pathogenesis of autoimmune diseases, such as RA, SLE, SS and SSc, has been demonstrated by both human and animal studies (104). As a crucial actor in maintaining immune system functions, the gut microbiota actively participates in T cell metabolic reprogramming (105). Many of these effects are mediated by metabolites produced by the microbes such as short-chain fatty acids (SCFAs, particularly the acetate, propionate and butyrate), bile acids (BAs), and tryptophan (Trp), as well as by the transformation of environmental or host molecules (106, 107) (**Figure 4**).

The internal balance of fatty acids can also exhibit significant effects on CD4+ T cells, notably regarding the generation of Th17 and Treg cells (108). SCFAs produced by the gut microbiota metabolizing dietary fibers can diffuse into the cytoplasm and serve as a substrate for FAO, further producing acetyl-CoA, which provides fuel for TCA and OXPHOS. However, fatty acids promote T cell differentiation into effector or regulatory T cells depending on immunological milieu. It is well appreciated that SCFAs promote the development of extrathymic Treg cells *via* induction of Foxp3 in a histone deacetylase (HDAC) dependent manner, while LCFAs contribute to the differentiation of pro-inflammatory Th1 and Th17 cells *via* the MAPKs p38 and JNK1 (109–112). In this setting, LCFA-rich diets have been confirmed to aggravate T cell-mediated autoimmune pathological process in mice, conversely, mice fed with SCFA are protected (112). Withal, studies indicated that addition of SCFAs such as acetate, propionate, and butyrate can increases intestinal IgA production, cytokine and chemokine production as well as mediate immune responses (113, 114). The mechanism is also related to the regulatory function of mTOR on immune cells (110, 111, 115). SCFAs act on production pathways of energy and induce ATP production and AMP consumption, which are inhibitors and activators of AMPK, respectively. Thus, the inhibitor activity of AMPK on mTOR is repressed, leading to mTOR activation in B cell (116, 117).

BAs also have an essential impact on T cells differentiation. In effector T cells, the derivative of lithocholic acid (LCA), isoalloLCA, stimulates OXPHOS and the production of mitochondrial reactive oxygen species (mitoROS), which leads to the upregulation of FOXP3 through histone (H3K27) acetylation in Foxp3 promoter region, resulting in Treg differentiation. Conversely, another derivative of LCA, 3-oxoLCA, inhibits the differentiation of Th17 cells by directly interacting with the key transcription factor retinoid-related orphan receptor-γt (RORγt) (118). In addition, BAs act through the BA receptor Breg to regulate the function of RORg+ Treg cells, which plays a pivotal role in maintaining the stability of the colonic environment (119). These studies indicate that BA and its metabolites regulate Th17 and Treg cell balance and elucidate the mechanism by which gut microbes govern the host immune response.

**Figure 5:**
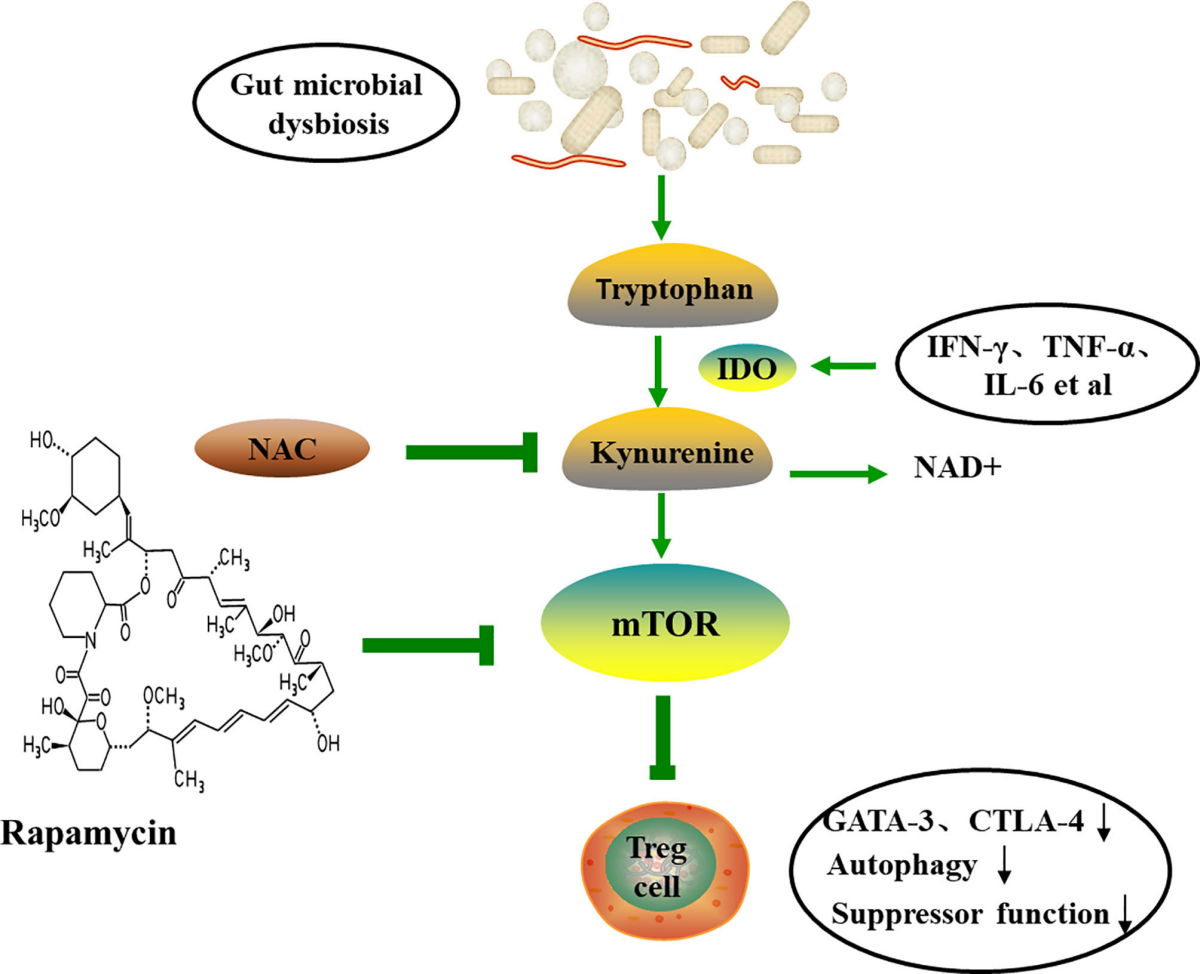
The tryptophan-kynurenine pathway and autoimmune. Trp can be metabolized to Kyn by IDO, and eventually lead to the generation of the NAD+ and the activation of mTOR. Therapeutic intervention with NAC reverses the accumulation of Kyn and the activation of mTOR, and induces autophagy and restores Treg functions. TNF-α, tumor necrosis factor-α; IFN-γ, interferon-γ; IDO, indoleamine-pyrrole 2,3-dioxygenase; NAC, N-acetylcysteine; NAD+, nicotinamide adenine dinucleotide; CTLA-4, cytotoxic T-lymphocyte-associated protein 4.

In addition, the Trp catabolism plays an important role in the peripheral immune tolerance by preventing autoimmunity. Gut microbiome dysregulation induces autoimmunity in lupus-susceptible mice by altering Trp metabolism (120). The kynurenine (Kyn) pathway is the major catabolic route of peripheral Trp metabolism, which accounts for >90% in mammals (121). Trp be metabolized to Kyn in the presence of indoleamine-pyrrole 2,3-dioxygenase (IDO), an alternative inducible enzyme, which is induced by pro-inflammatory cytokines, such as TNF-α, IL-6 and IFN-γ, and eventually bring about the generation of the nicotinamide adenine dinucleotide (NAD+) (122, 123). The increased Kyn was observed in lupus-prone mice (120). mTOR, a sensor of T cell mitochondrial homeostasis and regulator of T cell differentiation, can be activated by Kyn *in vitro*. A recent study revealed that PPP was the most significant metabolome changes in peripheral blood lymphocytes of SLE patients by mass spectrometry. In addition, the accumulation of Kyn was related to the increase of PPP activity, and N-acetylcysteine (NAC), a precursor of glutathione, significantly reduced the level of Kyn and also reduces disease activity in SLE patients by inhibiting mTOR (124). The study by Lai et al. showed that sirolimus, the inhibition of mTOR, increased CD4+CD25+FoxP3+ Treg cells and CD8+ memory T cells and restrained IL-4 and IL-17 production by CD4 and CD4-CD8- double-negative T cells (125). Mitochondrial metabolism, including mitochondrial hyperpolarization, ATP production and oxidative stress, play important roles in the abnormal activation of SLE T cells (126). mTOR is widely thought to be involved in the inhibition of autophagy and can be regulated with rapamycin (127). Furthermore, study has indicated that the expression of GATA-3 and CTLA-4 in SLE Treg cells is reduced and the autophagy and inhibitory functions are reduced. Rapamycin can induce autophagy, restore the expression of GATA-3 and CTLA-4, and correct Treg functions (**Figure 5**) (128). Mitochondrial autophagy is caused by the dynamin-related protein 1 (Drp1), which leads to mitochondrial degradation. The study by Caza et al. demonstrated that Ras-related protein 4-mediated loss of Drp1 leads to mitochondrial accumulation and disease progression in lupus-susceptible mice (126). In conclusion, the abnormal Trp metabolic caused by gut microbial disorder may be one of the mechanisms of autoimmune activation. Also, the butyrate produced by gut microbes exert anti-inflammatory function by promoting differentiation of Treg cell (129). All in all, probiotics may be an option for the prevention and treatment of autoimmune diseases.

### Single-Cell Immunometabolism of Treg/Th17 Cell

As every cell resides in a unique environment, no cell within our body is completely identical metabolically, phenotypically, and functionally, even of the same type, assessing the metabolic state of single immune cells becomes the focus of autoimmune disease (130). Meanwhile, the complexity of immune cells, the variety of different cell types in tissue, and heterogeneous clinical presentation make single-cell technologies a necessary means to uncover role of single cells (131). Recent years, the ability to extract and recognize biologically informative from single cell is improved with developments in the fields of machine learning. The field of immunology is dominated by high dimensional single-cell analysis, consisting of the first s sing-cell DNA sequencing based genomes reported in 2006, to the first realization of single-cell RNA-sequencing (scRNA-seq) based transcriptomics by Tang et al. in 2009, along with the recent Mass Cytometry, Met-Flow and mass spectrometry (MS) imaging that allowed directly measure metabolism *in situ* (132–138). Among them, scRNA-seq is the most widely used, which can efficiently amplify mRNA in isolated single cells and then conduct high-throughput sequencing, aiming to capture the entire transcriptome without the need to select target genes in advance, which can effectively solve the problem of transcriptome heterogeneity of immune cells and provides a multidimensional assessment of the transcriptional status. scRNA-seq allows individual cells to be defined as distinct metabolic entities and delineates corresponding transcripts from a metabolic perspective.

Caublomme et al. sequenced the transcriptome of 722 Th17 cells and validated four key molecules, Gpr65, Plzp, Toso and Cd5l, that governing the pathogenicity and susceptibility of autoimmune encephalomyelitis by calculating and analyzing a spectrum of cellular states *in vivo* to *in vitro* differentiated Th17 cells. Amongst them, Cd5l and Gpr65 associated with known regulatory and pro-inflammatory genes (139). Wagner has recently investigated metabolic differences associated with pathogenic/non-pathogenic Th17 cells by Compass, an algorithm to characterize the metabolic state of cells and relate metabolic heterogeneity to other cellular phenotypes based on single-cell RNA-Seq and flux balance analysis (140). This study also pinpointed the pathogenic Th17 cells is dependent on higher aerobic glycolysis and TCA to produce ATP, while nonpathogenic Th17 cells mainly rely on FAO for energy. The glycolytic enzyme phosphoglycerate mutase (PGAM) inhibits the pathogenicity of Th17 cell, and inhibition of G6PD alleviates neuroinflammation mediated by Th17 *in vivo*. Miragaia highlighted the metabolic heterogeneity among Tregs in colon, skin, spleen and lymph nodes of colon and skin in mouse by single-cell RNA-seq (141). In addition, a study based on Met-Flow found that metabolic remodeling reshaped the phenotype and function of T-cell subsets. Met-Flow, a high-dimensional flow cytometry approach, allows simultaneous analysis the metabolic state of key proteins and activation molecules on a single-cell in their representative pathways (142). This study also indicated that the expression of CD25, a characteristic surface marker molecule of Treg cells, is dependent on glycolysis and positively correlated with GLUT1 protein level.

Met-Flow overcomes inherent disadvantage of temporal inconsistency between mRNA abundance and protein concentration in traditional metabolic mRNA analysis (143, 144). The establishment of single-cell profiling technologies allows capturing the complex metabolic state of any cell, leading to a greater understanding of the role of metabolic heterogeneity in immune responses, which has taken the field of immunometabolism to a new level. Yet it is clear that additional effort are required to fully grasp the immunometabolism of Th17/Treg cell and for tasks such as metabolic clustering on the single-cell level.

## Metabolic Abnormalities on TH17/TREG Cell in Autoimmune Disease

Autoimmune disease is characterized by the imbalance in the immunomodulatory network (the destruction of balance such as Th1/Th2 and Th17/Treg cells) and activation or delayed apoptosis of immune cells. A balance between immune tolerance and inflammation is required to maintain optimal immunity and disruption of the Th17/Treg cell axis may contribute to the occurrence and development of disease. Studies have demonstrated that the expansion of Th17 cells and reduction of the suppression function and number of Treg represent the main causes in various AID (31–33, 100). Since metabolism controls the fate and function of immune cells, immunometabolism emerged as an active research field of multiple disease conditions and evidence in support of the metabolic differences between RA and SLE is accumulating (145–147).

Studies have demonstrated that RA T cells exhibit diminished glycolytic activity, reduced mitochondrial activity and low ATP production and low intracellular ROS concentrations, which deprives them of the energy required to generate oxidative stress or to execute autophagy, thus rendering them more inclined to utilize the PPP for production of NADPH (148, 149). These changes in metabolic status may be due to AMPK activity was significantly inhibited in RA T cells (150). The increase in mTORC1 activity in osteoclasts from patients with RA was also demonstrated (151). Additional evidence of the benefits of mTORC1 inhibition in RA includes the IL-17 inducing mTORC1 dependent proliferation of fibroblastlike synoviocytes (FLS) (152). In this context, it was demonstrated that the mTOR inhibitor rapamycin decreased the invasive properties of RA FLS (153). Furthermore, lactate, a breakdown product of glucose and dominant metabolite of the inflamed joint, induces IL-17 expression through nuclear PKM2 and FAS mediated STAT3 phosphorylation, thus affecting the effector function of T-cell (154). FAS has been implicated in the production of pathogenic Th17 cells, and blocking FAS suppressed the tissue invasion ability of T cells in RA (155).

Unlike RA T cells, CD4 T cells in SLE patients are committed to rapid conversion of nutrients into energy, which is manifested by high glycolysis activity, overuse of mitochondrial activation and excessive oxidative stress (156–158). A study have demonstrated that CD4+T cell glycolysis and mitochondrial oxidative metabolism were elevated in SLE patient and mice. Furthermore, 2-Deoxy-Dglucose (2DG) and metformin, the glucose metabolism inhibitor and mitochondrial metabolism inhibitor, reduced IFN-γ expression and normalized T cell metabolism, thereby reversing the disease (156). Overexpression of pyruvate dehydrogenase phosphatase catalytic subunit 2 (PDP2) weaken Th17 differentiation in SLE patients, whereas high levels of inducible cAMP early repressor (ICER) suppress PDP2 expression and ultimately decompose glucose into lactate (159). In addition, glutaminolysis as energy source plays a vital role in all proliferating cells. Kono. M et al. showed that ICER directly binds to glutaminase 1 (Gls1) to accelerate the glutaminolysis, thus promoting the differentiation of Th17 cells (160). Another study they performed also showed that inhibition of Gls1 affected glycolysis in MRL/lpr mice and ameliorated lupus-like disease and EAE by decreasing HIF-1α in Th17 cells (161). Sustained activation of mTORC1 is a metabolic feature shared by RA and SLE T cells. Overactivation of mTORC1 has been ascertained as a key factor in SLE T cell dysfunction. Accordingly, the mTOR inhibitor rapamycin inhibits pro-inflammatory T cell differentiation and thus plays an indispensable role in the amelioration of SLE (162). Additionally, increased mitochondrial metabolism is a major metabolic feature of Treg cells. Lack of mitochondrial respiratory chain complex III lead to a loss of suppressive capacity of Treg cell, thereby leading to the occurrence of inflammatory disease, but Foxp3 expression and quantity of Treg cell are not affected (163). The mitochondrial function of T cells in RA patients is defective, whereas the mitochondria and ROS of T cells in SLE patients are abundant (164). Taken together, T cells in different disease possess discrepant metabolic characteristics, which providing further support for the targeted treatment of the disease.

## Conclusion

Extracellular signals through regulating metabolic pathway drive T cell proliferation and differentiation. Metabolic pathways for the effects of T cell development and function have become the focus of immunology research. This paper reviews the metabolic characteristics, physiological functions and regulatory mechanisms of Th17 and Treg cells, which can help to elucidate the immune metabolic pathways of chronic inflammatory conditions. It must be stressed, however, further research is needed to better understand the pathways of immunometabolism and thus provide new insights into the pathogenesis and treatment of the autoimmune diseases.

## Author Contributions

YQ developed and wrote the review. CG provided significant revisions to the manuscript. JL generated themes, guided, and edited the manuscript. All authors contributed to the article and approved the submitted version.

## Funding

This work was supported by the Nature Fund Projects of Shanxi Science and Technology Department (201901D111377), the Scientific Research Project of Health commission of Shanxi Province (2019044), the Research Project Supported by Shanxi Scholarship Council of China (2020-191) and Science and Technology Innovation Project of Shanxi Province (2020SYS08).

## Conflict of Interest

The authors declare that the research was conducted in the absence of any commercial or financial relationships that could be construed as a potential conflict of interest.

## Publisher’s Note

All claims expressed in this article are solely those of the authors and do not necessarily represent those of their affiliated organizations, or those of the publisher, the editors and the reviewers. Any product that may be evaluated in this article, or claim that may be made by its manufacturer, is not guaranteed or endorsed by the publisher.
